# Topographic Evaluation of Unilateral Keratoconus Patients

**DOI:** 10.4274/tjo.galenos.2018.90958

**Published:** 2019-06-27

**Authors:** Cumali Değirmenci, Melis Palamar, Nergis İsmayilova, Sait Eğrilmez, Ayşe Yağcı

**Affiliations:** 1 Ege University Faculty of Medicine, Department of Ophthalmology, İzmir, Turkey; 2 Dünyagöz Hospital, Ophthalmology Clinic, Baku, Azerbaijan

**Keywords:** Amsler-Krumeich, Scheimpflug camera, unilateral keratoconus

## Abstract

**Objectives:**

To compare data obtained by Scheimpflug camera (Pentacam) from both eyes of unilateral keratoconus patients and normal controls.

**Materials and Methods:**

This study was performed by retrospective chart review of 919 keratoconus patients. From these patients, 31 keratoconus eyes of 31 patients with unilateral keratoconus (Group 1), 31 normal fellow eyes of these patients (Group 2), and 30 right eyes of 30 normal controls (Group 3) were included in the study. Detailed ophthalmologic examination and Pentacam parameters at initial examination were analyzed and relationships between Groups 1, 2, and 3 were statistically evaluated. ROC curve analysis was also performed to determine the sensitivity and specificity of parameters that could be used to differentiate Group 2 from Groups 1 and 3.

**Results:**

The mean age was 30.07±11.00 (15-60) in Group 1-2 patients and 32.33±9.30 (18-45) in Group 3 patients (p=0.392). In comparison of Pentacam data, there were statistically significant differences between Groups 1 and 2 in all parameters except corneal volume (p<0.05). Group 1 and Group 3 were significantly different in all evaluated parameters (p<0.05). Steep keratometry, flat keratometry, mean keratometry, and posterior elevation (PE) were statistically similar between Groups 2 and 3 (p>0.05), while the other evaluated parameters differed significantly (p<0.05). ROC curve analysis showed that the difference in corneal thickness between the apex and thinnest point, progression index, index of surface variance, index of height asymmetry and inferior-superior had the highest sensitivity and specificity in differentiating Group 2 from Group 3, while CCTapex, CCTmin, PE, and minumum radius had the highest sensitivity and specificity in differentiating Group 2 from Group 1.

**Conclusion:**

In patients with unilateral keratoconus, fellow eyes appear to not be completely normal. Thus, it is recommended that fellow eyes also be evaluated in every examination of unilateral keratoconus patients.

## Introduction

Keratoconus is a progressive corneal disease characterized by central corneal thinning, high myopia, and irregular astigmatism. The incidence of keratoconus is approximately 1/2000 and its prevalence is 54.5/100,000. The disease is caused by both genetic and environmental factors.^1,2,3,4^

In addition to clinical examination, various auxiliary instruments are used in the diagnosis of keratoconus. In the past, keratoconus was diagnosed using Placido-disc based topographers, which are only able to evaluate the anterior surface of the cornea. The development of the Scheimpflug camera system (Pentacam, Oculus Optikgerate GmbH, Wetzlar, Germany) also enabled evaluation of the posterior cornea surface. This device allowed the detection of early changes originating in the posterior cornea in clinically normal patients, which was a major breakthrough in the diagnosis and monitoring of the disease.^5,6,7^

Keratoconus is usually progressive and bilateral. Even if one eye is not affected initially, the fellow eye is eventually affected as well in the majority of patients. Holland et al.^8^ determined that 50% of patients initially diagnosed with unilateral keratoconus also developed keratoconus in the apparently normal fellow eye. However, Imbornoni et al.^9^ emphasized that keratoconus was not observed during long-term follow-up in any of the fellow eyes in a series of 5 cases. Therefore, different terms such as preclinical keratoconus, forme fruste keratoconus, and keratoconus suspect are used instead of unilateral keratoconus.^5,10,11^ Although different rates have been reported for unilateral keratoconus, the proportion generally ranges between 0.5% and 4.5%.^8,12,13,14,15,16^ Various keratoconus studies have demonstrated abnormalities in the Pentacam data of fellow eyes considered unaffected.^2,11,17,18,19^

The aim of this study was to compare anterior segment parameters of the apparently normal fellow eyes of patients who presented to our center with unilateral keratoconus with those of keratoconus eyes and the eyes of healthy control subjects.

## Materials and Methods

This study was carried out with the approval of the Ege University Faculty of Medicine Ethics Committee (129362). The medical data of patients with keratoconus who presented to the Cornea Unit of the Ege University Faculty of Medicine Department of Ophthalmology were retrospectively analyzed. Patients who had a history of trauma or corneal surgery for keratoconus and those from whom reliable measurements could not be obtained were not included in the study. In addition, patients who used contact lenses at time of presentation and those with a history of allergic conjunctivitis were excluded from the study. Of the remaining 919 patients, 31 patients (3.3%) who had been evaluated as having unilateral keratoconus at initial presentation were included in the study. The patients’ best corrected visual acuity, intraocular pressure measurements, and anterior and posterior segment examination findings were evaluated. In addition, the patients’ keratometric parameters, topometric parameters, posterior elevation, corneal pachymetry, and pachymetric index values obtained with Pentacam were analyzed.

The eyes were divided into 3 groups: keratoconus eyes (Group 1, 31 eyes of 31 patients), fellow eyes considered clinically and topographically normal (Group 2, 31 eyes of 31 patients), and the healthy right eyes of control subjects (Group 3, 30 eyes of 30 patients). The Amsler-Krumeich classification was used when diagnosing keratoconus.^20^ According to this classification, stage 1 is defined as eccentric steepening, myopia and/or astigmatism <5 D, and/or central keratometry value <48 diopter (D); stage 2 involves myopia and/or astigmatism of 5-8 D, central keratometry value <53 D, and minimum corneal thickness >400 µm; stage 3 is defined as myopia and/or astigmatism of 8-10 D, central keratometry value >53 D, and minimum corneal thickness 300-400 µm; and in stage 4, refraction is not measurable, central keratometry value is >55 D, there is central corneal scarring and minimum corneal thickness is <200 microns. The groups were compared in terms of demographic and Pentacam (Oculus Optikgerate GmbH, Wetzlar, Germany) data. Measurements were repeated until a reliable measurement was obtained according to the Pentacam device’s software. Our analysis included the following Pentacam data: the anterior corneal surface keratometric parameters steep keratometry (Ks), flat keratometry (Kf), mean keratometry (Km), and the inferior-superior (I-S) difference at 4 mm; the topometric parameters index of surface variance (ISV), index of vertical asymmetry (IVA), keratoconus index (KI), central keratoconus index (CKI), index of height asymmetry (IHA), index of height decentration (IHD), and minimum radius (Rmin); and posterior elevation (PE), corneal thickness at the apex and the thinnest point (CCTapex, CCTmin), corneal volume (CV), and mean pachymetric progression index (PPI).

### Statistical Analysis

Statistical analyses were done using the SPSS software package version 20 (IBM Corp., 2011). The Shapiro–Wilk test was used to test all parameters for normal distribution. Comparisons between groups were done with one-way ANOVA with post-hoc Bonferroni test. Chi-square test was used to compare demographic data. ROC curve analysis was done to determine the sensitivity and specificity of the parameters. A p value below 0.05 was considered statistically significant.

## Results

**Figure 1:**
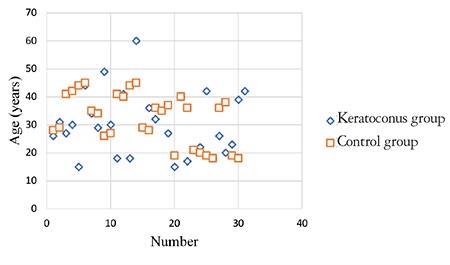
Age distribution curves of the groups

Mean age was 30.07±11.00 (15-60) years in Groups 1/2 and 32.33±9.30 (18-45) years in Group 3 (p=0.392) (Figure 1). The female to male ratio was 11/19 in Groups 1/2 and 16/14 in Group 3 (p=0.194).

Comparison of Pentacam data between Groups 1 and 2 showed that Ks, Kf, Km, PE, I-S difference, ISV, IVA, KI, CKI, IHA, IHD, and PPI values were significantly higher in Group 1 (p<0.05) (Table 1). In contrast, Rmin, CCTapex, and CCTmin were significantly lower in Group 1 (p<0.05), while CV was similar between the groups (p=0.383).

In comparisons of Groups 1 and 3, Group 1 had significantly higher Ks, Kf, Km, PE, I-S difference, ISV, IVA, KI, CKI, IHA, IHD, and PPI (p<0.001) and significantly slower Rmin, CCTapex, and CCTmin (p<0.001). CV was also significantly lower in Group 1 than in Group 3 (p=0.009).

Comparisons of Groups 2 and 3 revealed similar Ks, Kf, Km, and PE (p=0.139, 0.473, and 0.239, respectively). The other analyzed parameters (I-S difference, ISV, IVA, KI, CKI, IHA, IHD, PPI, Rmin, CCTapex, CCTmin, and CV) all differed significantly between the two groups (p<0.05).

**Figure 2:**
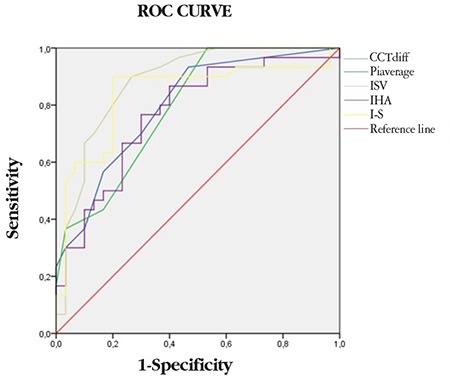
ROC curve between normal fellow eyes of keratoconus patients and healthy controls I-S: Inferior-superior, IHA: Index of height asymmetry, ISV: Index of surface variance, CCT: Central corneal thickness

**Figure 3:**
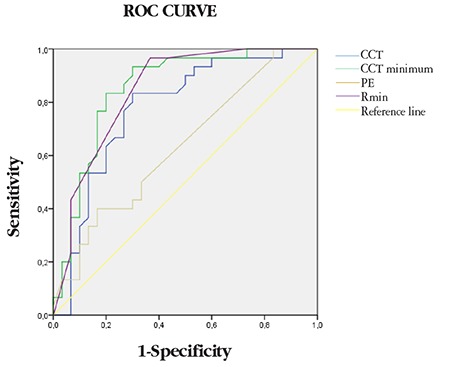
ROC curve between the keratoconus eyes and normal fellow eyes of keratoconus patients CCT: Central corneal thickness, PE: Posterior elevation, Rmin: Minimum radius

In ROC curve analysis to identify parameters that could be used to differentiate Groups 2 and 3, the parameters with the largest areas under the curve (AUC) were ISV (threshold 18.50, AUC=0.88) and I-S (threshold 1.25, AUC=0.84). In addition, PPI, IHA, and CCTdiff, which is the difference between CCTapex and CCTmin, also had significantly high AUC values (Table 2, Figure 2). ROC curve analysis between the eyes in Groups 1 and 2 aimed to differentiate the apparently normal fellow eyes in Group 2 from keratoconus eyes and showed that CCTapex, CCTmin, PE, and Rmin had high sensitivity and specificity in the differentiation of Group 2 from Group 1 (Table 3 and Figure 3).

## Discussion

Keratoconus is a chronic, usually bilateral, non-inflammatory corneal ectasia.^1^ The corneal thinning seen in keratoconus is not central, but usually occurs in the inferonasal region. The Pentacam has a key role in the early diagnosis and monitoring of keratoconus due to its ability to evaluate the anterior and posterior corneal surfaces together. Abnormalities emerge in the posterior surface of the cornea in early keratoconus. Therefore, development of the Pentacam led to a significant increase in diagnostic sensitivity in keratoconus.^1,2,4^

In this study, we attempted to identify differences between the apparently normal fellow eyes of patients with unilateral keratoconus and the patients’ keratoconus eyes and the healthy eyes of controls. Comparison of Pentacam data revealed significant differences between Groups 1 and 2 in all parameters except CV. Çağıl et al.^21^ compared CV in keratoconus patients, subclinical keratoconus patients, and normal control subjects and showed that this parameter is helpful in distinguishing keratoconus eyes from normal eyes but not in differentiating between keratoconus and subclinical keratoconus. In their study of patients with keratoconus, Emre et al.^22^ found that CV decreased with disease progression. The results of our study also support these data. We found that the keratoconus eyes in Group 1 differed significantly from the eyes in Groups 2 and 3. We also determined that the Pentacam data of the eyes in Group 2 were statistically closer to the results in Group 3, especially in terms of keratometry readings. In another study on this subject, Bae et al.^17^ compared the affected and unaffected eyes of patients with keratoconus and reported a significant difference, with normal fellow eyes being more similar to the eyes of healthy volunteers. Hashemi et al.^23^ found that the normal fellow eyes of patients with keratoconus were not significantly different in terms of average keratometry values, but did show significant differences in topometric indexes. These findings are consistent with the data obtained in the current study. These results may be due to evaluating the patients’ fellow eyes before emergence of the disease or to the patients having true unilateral keratoconus. However, in contrast to these studies, Muftuoglu et al.^11^ found that the fellow eyes of patients with keratoconus were significantly different from healthy controls. Further studies are needed to be able to clearly differentiate these eyes.

In this study, we also performed ROC curve analysis to enable the discrimination of clinically unaffected fellow eyes of keratoconus patients from keratoconus eyes and eyes of healthy subjects. According to our results, CCTdiff, PPI, ISV, IHA, and I-S showed high sensitivity and specificity for distinguishing Group 2 from Group 3, while CCTapex, CCTmin, PE, and Rmin values showed high sensitivity and specificity for differentiating between Group 2 and Group 1. In their study, Muftuoglu et al.^11^ found that I-S and PPI had high sensitivity and specificity for discriminating keratoconus patients from healthy controls. Bae et al.^17^ mentioned the importance of I-S and PE in evaluating the fellow eyes of patients with keratoconus in their study, while Mihaltz et al.^24^ emphasized that PE was the most sensitive parameter for diagnosing keratoconus. Hashemi et al.^23^ reported that in addition to pachymetric indices, IVA and ISV showed high accuracy rates in identifying cases of subclinical keratoconus. The results obtained in the present study are also consistent with the aforementioned studies. However, none of these values alone is sufficient for the diagnosis or discrimination of keratoconus. Combining them with other clinical data may increase their diagnostic value.

### Study Limitations

Limitations of this study include its retrospective nature, the limited number of subjects, and the fact that conclusions were based solely on measurements made at the time of presentation.

## Conclusion

To summarize, the results of this study indicate that although the fellow eyes of patients diagnosed with unilateral keratoconus did not exhibit measurement anomalies great enough to be considered keratoconic at the time of diagnosis, they were also not completely normal. However, based on the data obtained, it does not seem possible to diagnose these eyes with keratoconus using available diagnostic tests. In patients with unilateral keratoconus, it is particularly important to monitor fellow eyes evaluated as normal at presentation for development of keratoconus in the long term and to advise patients to avoid mechanical trauma.

## Figures and Tables

**Table 1:**
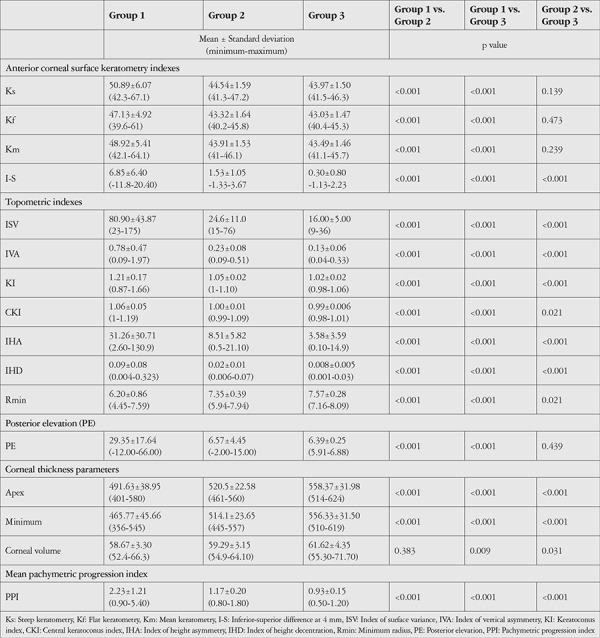
Mean values of anterior segment parameters and comparisons between groups

**Table 2:**
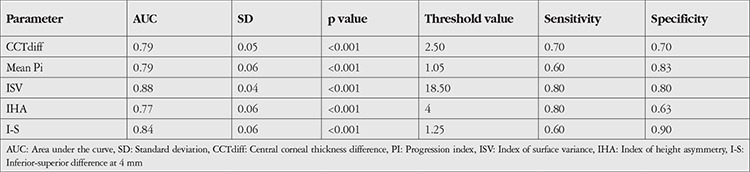
ROC curve analysis for discriminating normal fellow eyes of unilateral keratoconus patients from eyes of the control group

**Table 3:**
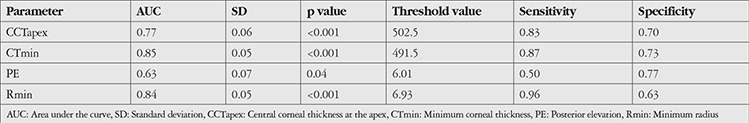
ROC curve analysis for discrimination of the keratoconus eyes and normal fellow eyes of patients with unilateral keratoconus

## References

[1] Rabinowitz YS (1998). Keratoconus. Surv Ophthalmol..

[2] Kamiya K, Ishii R, Shimizu K, Igarashi A (2014). Evaluation of corneal elevation, pachymetry and keratometry in keratoconic eyes with respect to the stage of Amsler-Krumeich classification. Br J Ophthalmol..

[3] Palamar M, Onay H, Ozdemir TR, Arslan E, Egrilmez S, Ozkinay F, Yagci A (2014). Relationship between IL1beta-511C>T and ILRN VNTR polymorphisms and keratoconus. Cornea..

[4] Gordon-Shaag A, Millodot M, Shneor E, Liu Y (2015). The genetic and environmental factors for keratoconus. Biomed Res Int..

[5] Kovacs I, Mihaltz K, Kranitz K, Juhasz E, Takacs A, Dienes L, Gergely R, Nagy ZZ (2016). Accuracy of machine learning classifiers using bilateral data from a Scheimpflug camera for identifying eyes with preclinical signs of keratoconus. J Cataract Refract Surg..

[6] Gokul A, Vellara HR, Patel DV (2018). Advanced anterior segment imaging in keratoconus: a review. Clin Exp Ophthalmol..

[7] Belin MW, Khachikian SS (2009). An introduction to understanding elevationbased topography: how elevation data are displayed - a review. Clin Exp Ophthalmol..

[8] Holland DR, Maeda N, Hannush SB, Riveroll LH, Green MT, Klyce SD, Wilson SE (1997). Unilateral keratoconus. Incidence and quantitative topographic analysis. Ophthalmology..

[9] Imbornoni LM, Padmanabhan P, Belin MW, Deepa M (2017). Long-Term Tomographic Evaluation of Unilateral Keratoconus. Cornea.

[10] Klyce SD (2009). Chasing the suspect: keratoconus. Br J Ophthalmol..

[11] Muftuoglu O, Ayar O, Ozulken K, Ozyol E, Akıncı A (2013). Posterior corneal elevation and back difference corneal elevation in diagnosing forme fruste keratoconus in the fellow eyes of unilateral keratoconus patients. J Cataract Refract Surg..

[12] Lee LR, Hirst LW, Readshaw G (1995). Clinical detection of unilateral keratoconus. Aust N Z J Ophthalmol..

[13] Phillips AJ (2003). Can true monocular keratoconus occur?. Clin Exp Optom.

[14] Rabinowitz YS, McDonnell PJ (1989). Computer-assisted corneal topography in keratoconus. Refractive Corneal Surg..

[15] Rabinowitz YS, Nesburn AB, McDonnell PJ (1993). Videokeratography of the fellow eye in unilateral keratoconus. Ophthalmology..

[16] Wilson SE, Lin DT, Klyce SD (1991). Corneal topography of keratoconus. Cornea..

[17] Bae GH, Kim JR, Kim CH, Lim DH, Chung ES, Chung TY (2014). Corneal topographic and tomographic analysis of fellow eyes in unilateral keratoconus patients using Pentacam. Am J Ophthalmol..

[18] Orucoglu F (2013). Incidence and Tomographic Evaluation of Unilateral Keratoconus. Turk J Ophthalmol..

[19] Aksoy S, Akkaya S, Özkurt Y, Kurna S, Açıkalın B, Şengör T (2017). Topography and Higher Order Corneal Aberrations of the Fellow Eye in Unilateral Keratoconus. Turk J Ophthalmol..

[20] Krumeich JH, Kezirian GM (2009). Circular keratotomy to reduce astigmatism and improve vision in stage I and II keratoconus. J Refract Surg..

[21] Çağıl N, Çakmak HB, Uğurlu N, Kocamış Sİ, Simavlı H, Şimşek Ş (2013). Corneal Volume Measurements with Pentacam for Detection of Keratoconus and Subclinical Keratoconus. Turk J Ophthalmol..

[22] Emre S, Doganay S, Yologlu S (2007). Evaluation of anterior segment parameters in keratoconic eyes measured with the Pentacam system. J Cataract Refract Surg..

[23] Hashemi H, Beiranvand A, Yekta A, Maleki A, Yazdani N, Khabazkhoob M (2016). Pentacam top indices for diagnosing subclinical and definite keratoconus. J Curr Ophthalmol..

[24] Mihaltz K, Kovacs I, Takacs A, Nagy ZZ (2009). Evaluation of keratometric, pachymetric, and elevation parameters of keratoconic corneas with pentacam. Cornea..

